# Water immersion methods do not alter muscle damage and inflammation biomarkers after high-intensity sprinting and jumping exercise

**DOI:** 10.1007/s00421-020-04481-8

**Published:** 2020-09-02

**Authors:** E. K. Ahokas, H. Kyröläinen, A. A. Mero, S. Walker, H. G. Hanstock, J. K. Ihalainen

**Affiliations:** 1 Biology of Physical Activity, NeuroMuscular Research Center, Faculty of Sport and Health Sciences, University of Jyväskylä, P.O Box 35, 40014 Jyväskylä, Finland; 2 Swedish Winter Sports Research Centre, Department of Health Sciences, Mid Sweden University, Östersund, Sweden

**Keywords:** Cold-water immersion, Thermoneutral water immersion, Contrast water therapy, Recovery, Inflammation

## Abstract

**Purpose:**

The aim of this study was to compare the efficacy of three water immersion interventions performed after active recovery compared to active recovery only on the resolution of inflammation and markers of muscle damage post-exercise.

**Methods:**

Nine physically active men (*n* = 9; age 20‒35 years) performed an intensive loading protocol, including maximal jumps and sprinting on four occasions. After each trial, one of three recovery interventions (10 min duration) was used in a random order: cold-water immersion (CWI, 10 °C), thermoneutral water immersion (TWI, 24 °C), contrast water therapy (CWT, alternately 10 °C and 38 °C). All of these methods were performed after an active recovery (10 min bicycle ergometer), and were compared to active recovery only (ACT). 5 min, 1, 24, 48, and 96 h after exercise bouts, immune response and recovery were assessed through leukocyte subsets, monocyte chemoattractant protein-1, myoglobin and high-sensitivity C-reactive protein concentrations.

**Results:**

Significant changes in all blood markers occurred at post-loading (*p *< 0.05), but there were no significant differences observed in the recovery between methods. However, retrospective analysis revealed significant trial-order effects for myoglobin and neutrophils (*p* < 0.01). Only lymphocytes displayed satisfactory reliability in the exercise response, with intraclass correlation coefficient > 0.5.

**Conclusions:**

The recovery methods did not affect the resolution of inflammatory and immune responses after high-intensity sprinting and jumping exercise. It is notable that the biomarker responses were variable within individuals. Thus, the lack of differences between recovery methods may have been influenced by the reliability of exercise-induced biomarker responses.

**Electronic supplementary material:**

The online version of this article (10.1007/s00421-020-04481-8) contains supplementary material, which is available to authorized users.

## Introduction

High-intensity exercise results in numerous physiological perturbations and insufficient recovery from these perturbations might result in suboptimal performance and training quality during subsequent training sessions, while chronic imbalance between training stress and recovery might lead to suboptimal training adaptations (Bleakly & Davison 2010). Water immersion methods are nowadays popular recovery methods among athletes as well as the general population. Immersion strategies can be divided into four major categories: cold-water immersion (CWI; ≤ 20 °C), hot water immersion (HWI; ≥ 36 °C), thermoneutral water immersion (TWI; 21‒35 °C) and contrast water therapy (CWT; alternating CWI and HWI) (Versey et al. 2013). These recovery interventions may be used with the intention of attenuating delayed onset of muscle soreness and accelerating recovery from muscle-damaging exercise (Pournot et al. 2011; Versey et al. 2013; Dupuy et al. 2018).

Although the majority of peer-review papers have observed limited positive effects of immersion compared to control groups for recovery measures, there is substantial heterogeneity in loading protocols and recovery responses. To this end, meta-analytical data suggests potential small but positive effects of CWI (Bleakley et al. 2012; Higgins et al. 2017; Dupuy et al. 2018) and CWT (Higgins et al. 2017) on performance and/or perceptual recovery from high-intensity loading exercise. Furthermore, muscle soreness may also be attenuated by CWI (Bleakley et al. 2012; Dupuy et al. 2018) and CWT (Dupuy et al. 2018). A number of mechanisms have been suggested to account for the enhanced acute recovery associated with post-exercise water immersions, one being a blunted inflammation response (White et al. 2014).

On the other hand, post-exercise inflammation may play an important role in mediating exercise-induced adaptations (Brunelli and Rovere-Querini 2008; Chazaud et al. 2009); for example, macrophages stimulate myoblast proliferation and differentiation, and thus are involved in skeletal muscle regeneration and tissue repair (Chazaud et al. 2009). Consequently, it has been suggested that water immersion methods could attenuate potentially desirable inflammatory responses to muscle-damaging exercise (Eston and Peters 1999; Vaile et al. 2007; Stacey et al. 2010; Murray and Cardinale 2015). Chronic use of CWI or other water immersion methods has been suggested to ultimately attenuate training adaptations (Yamane et al. 2006, 2015; Roberts et al. 2014).

The majority of previous studies have reported that CWI (Pournot et al. 2011; de Andrade Bezerra et al. 2014) and other water immersion methods (Pournot et al. 2011) do not affect leukocyte, lymphocyte, neutrophil, monocyte and macrophage counts compared to passive or active recovery. Nevertheless, Stacey et al. (2010) found that, an hour after exercise, leukocyte and neutrophil counts were higher and lymphocyte counts were lower after CWI compared to active and passive recovery, which could be beneficial for short-term recovery (Stacey et al. 2010). Furthermore, an animal study by Camargo et al. (2012) showed that the density of inflammatory cells (cell/100 μm^2^) in rats muscle tissue was lower after CWI than in passive recovery (Camargo et al. 2012).

There is a limited amount of research on the effects of water immersion methods on muscle damage and inflammation markers and conflicting results about the effect of the water immersion methods on leukocytes and leukocyte subsets. Therefore, the purpose of this study was to examine the effect of three different water immersion methods after active recovery and active recovery alone on leukocytes, leukocyte subsets, myoglobin, and monocyte chemoattractant protein-1 concentration and recovery following an acute bout of high-intensity exercise. The hypothesis was that water immersion methods after an active recovery do not give any added benefits on the clearance of muscle damage and inflammation markers following active recovery.

## Methods

### Participants

Nine healthy, men (mean ± SD: age 26 ± 3.7 y, body mass 78.6 ± 11.6 kg, height 1.81 ± 0.09 m, body fat: 15.8 ± 4.0%), undertaking regular physical activity, provided written, informed consent to take part in the study. Sample size was estimated based on previous studies (Stacey et al. 2010; Crystal et al. 2013; Broatch et al. 2014; Peake et al. 2017). Upon enrolment, participants completed a health questionnaire, as well as food and activity diaries that facilitated standardisation of nutritional intake and physical activity before, during, and after the trials until the last recovery measurements. Ethical approval for the study was obtained from the Ethical Committee of University of Jyväskylä, and the study was conducted in accordance with the Declaration of Helsinki.

### Study design and procedures

**Fig. 1:**
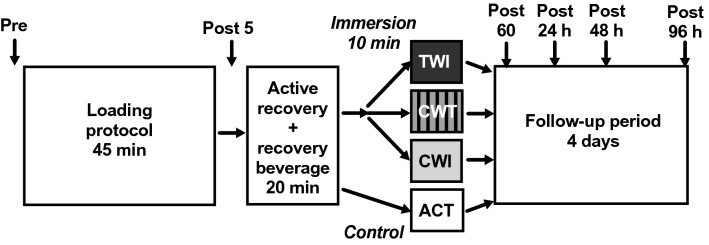
Schematic of study design. Participants completed a standardised loading protocol followed by an initial recovery protocol (active recovery and recovery beverage). After the active recovery protocol participants completed one of three water immersion protocols (*TWI* temperate water immersion, *CWT* contrast water therapy or *CWI* cold-water immersion) or sat in an empty bath (*ACT* active recovery only, serving as control). Follow-up measurements were obtained at four additional timepoints after immersion (marked by arrows)

The recovery protocol was designed to match typical recovery routines used by athletes and the purpose was to investigate the possibility of added benefit from water immersion methods compared to standard (active) recovery routines frequently used by athletes. A schematic representation of the protocol has been published elsewhere (Ahokas et al. 2019). Briefly, participants were randomly allocated to active recovery only (ACT), or active recovery followed by cold-water immersion (CWI), contrast water therapy (CWT) or thermoneutral water immersion (TWI) on each test day. All participants completed all four experimental conditions, separated by at least 2 weeks, each time with a different recovery method. Prior to the recovery intervention, participants performed several physical performance tests and an intensive loading protocol, which included jumping and sprinting. The recovery procedure included active recovery (10 min bicycle ergometer, Monark 839 E, Vansbro, Sweden; heart rate 120–140 bpm) accompanied by consumption of a recovery beverage (Gainomax, 40 g carbohydrates and 20 g protein) and followed the assigned recovery intervention. The fluid intake was controlled during the loading protocol and the first hour of recovery. White blood cells and differential cell counts, MCP-1 and biochemical markers of muscle damage were measured at baseline (PRE), and then at five timepoints following the loading protocol: 5 min, 1 h 24 h, 48 h, and 96 h post-loading (POST5, POST60, POST24, POST48 and POST96, respectively; Fig. 1).

### Loading protocol and performance tests

On each of the four trials, participants performed an intensive loading exercise protocol (total duration 45 min; Fig. 1), which included:

- 2 × 5 ×10 unilateral long jumps (walking back between sets / 5 min rest between blocks)
- 2 × 3 × 60 m running:
- Set 1: the target speed was 95% of the maximum speed tested the same morning;
- Set 2: the target speed was 98% of the maximum speed;
- 2 min rest between repetitions / 5 min rest between sets.
- 2 × 200 m run at maximum speed (5 min rest).

During exercise, heart rate was continuously recorded with a heart rate monitor (Polar V800, Kempele, Finland), and there were no differences between trials. The performance tests involved a single 30 m maximal sprint, maximum bilateral isometric leg press and countermovement jump. The performance test protocol has been reported elsewhere and there were no differences in performance test results following different immersions (Ahokas et al. 2019). Furthermore, creatine kinase (CK) and cortisol (COR) responses in relation to self-reported muscle soreness have been reported elsewhere (Ahokas et al. 2019), and are presented here in a posteriori reliability analyses.

### Water immersions

Following active recovery and consumption of the recovery beverage, each participant was immersed underwater in a sitting position to the level of the xiphoid process for 10 min in a dedicated bath, while during the ACT condition the participants remained seated in an empty bath for 10 min. CWI and TWI were continuously immersed in water temperatures of 10° and 24 °C, respectively. In CWT, the participants alternated immersion at 38 °C and 10 °C with five cycles of 1 min in each bath.

### Collection and analysis of blood samples

To assess the immediate and prolonged immune responses to each loading protocol/recovery protocol, blood samples were drawn from an antecubital vein into EDTA tubes (Venosafe, Terumo, Belgium), and serum tubes (Vacuette, Greiner-Bio-One GmbH, Kremsmünster, Austria) at baseline and at 5 min, 60 min, 24 h, 48 h and 96 h after completing the loading protocol. Total white blood cell count and subgroups; neutrophils, lymphocytes and mixed cells (monocytes, eosinophils, basophils and immature precursor cells) were determined with an automated cell counter (Sysmex KX-21 N, TOA Medical Electronics Co., Ltd., Kobe, Japan). Serum samples were held for 30 min at room temperature before being centrifuged for 10 min at 2000×*g* at 4 °C (Megafuge 1.0 R, Heraeus, Germany). Serum was then stored at −80 °C until analysis. Serum samples were analysed for high-sensitivity C-reactive protein (hs-CRP) using commercial chemiluminescence immunoassay techniques (Immulite 2000 XPi, Siemens Healthcare GmbH, Erlangen, Germany). Myoglobin and MCP-1 were analysed in serum samples in single using an enzyme-linked immunosorbent assay (ELISA) method with commercial reagents (R&D Systems, Europe Ltd, Abingdon, UK) performed on an automated platform (Dynex DS2, Washington, USA). The detection limits and inter-assay coefficients of variation, respectively, were 0.1 mg L^−1^ and 10.1% for hs-CRP, 0.8 ng mL^−1^ and 10.2% for myoglobin and 3.9 pg mL^−1^ 6.2% for MCP-1.

### Statistical analyses

Statistics were performed using commercial software packages (IBM SPSS version 24.0; SPSS Inc., Chicago, IL; Prism 8, GraphPad, La Jolla, CA). Data were checked for normality using Shapiro–Wilk tests; variables that did not satisfy the assumption of normality were log-transformed prior to analysis. Conventional statistical methods were used to obtain means, standard deviation (SD), 95% confidence intervals (95% CI), and Pearson’s product moment correlation coefficients; for log-normally distributed variables, geometric mean and SD factors are reported. Two-way repeated-measures analysis of variance (ANOVA) was used to compare continuous variables between conditions across the pre- and post-loading and immersion timepoints. Due to two missing data points in the leukocyte analyses, a mixed-effects model using the maximum likelihood method was performed instead of a two-way repeated-measures ANOVA. Post-hoc tests were used to explore time effects compared to baseline within trials using Dunnet’s correction for multiple comparisons. Data are reported and displayed as means and standard deviations (SD) for normally distributed data sets and geometric means and geometric SD for data sets that were log-transformed prior to analysis. Statistical significance was set at *p* ≤ 0.05 but trends were explored with post-hoc tests when *p* ≤ 0.1 for main effects.

## Results

### White blood cell responses to recovery

**Fig. 2:**
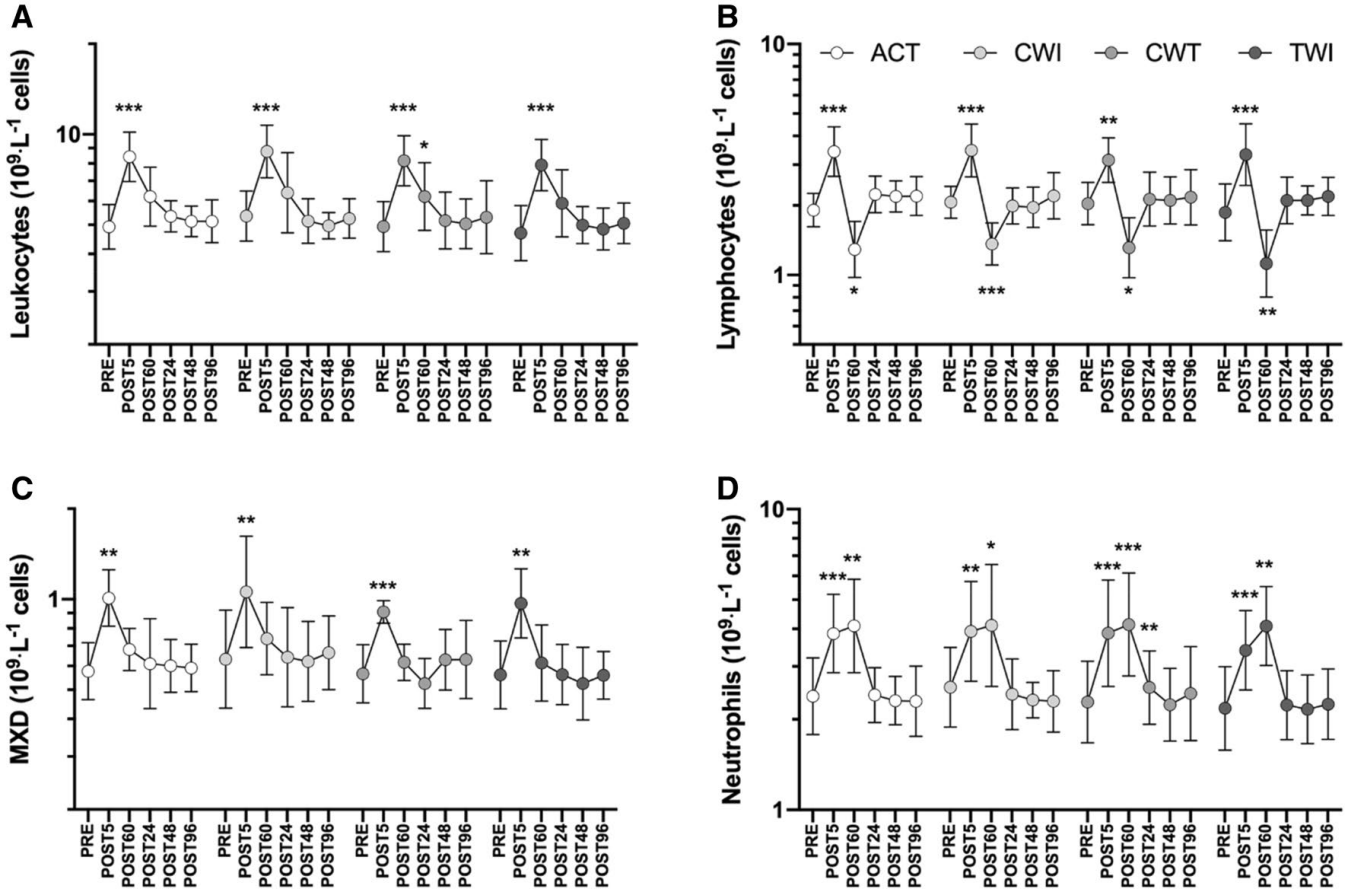
Leukocyte (**a**), lymphocyte (**b**), mixed cell (**c**; MXD; monocytes, eosinophils, basophils and immature precursor cells) and neutrophil (**d**) responses to the loading protocol and recovery. Recovery interventions applied between POST5 and POST60; *ACT* active recovery only, *CWI* cold-water immersion, *CWT* contrast water therapy and *TWI* thermoneutral water immersion. Data are geometric mean and SD factors displayed on a log_10_ scale. No differences in leukocyte or leukocyte subset responses were found between recovery interventions; however, effects of time are displayed for each subset as differences from PRE. *p < 0.05, **p < 0.01, ***p < 0.001

There were no differences in the magnitude of the exercise-induced leukocyte response between trials. Furthermore, there were no differences in leukocytes or leukocyte subset concentrations during recovery between recovery interventions (Fig. 2). As expected, multiple significant time effects were observed between timepoints for all variables, illustrated in Fig. 2; for clarity, only differences from PRE for each trial are displayed. Leukocytes and all subsets increased in response to the loading protocol (*p* < 0.01 for all variables, Fig. 2a). Leukocytes and mixed cells (Fig. 2c) generally returned to baseline around POST60, while lymphocytes (Fig. 2b) increased at POST5 then decreased to below PRE values at POST60. Neutrophils generally remained elevated until POST60 (Fig. 2d). All leukocyte subsets had returned to baseline by POST24.

### Biomarker responses to recovery

**Fig. 3:**
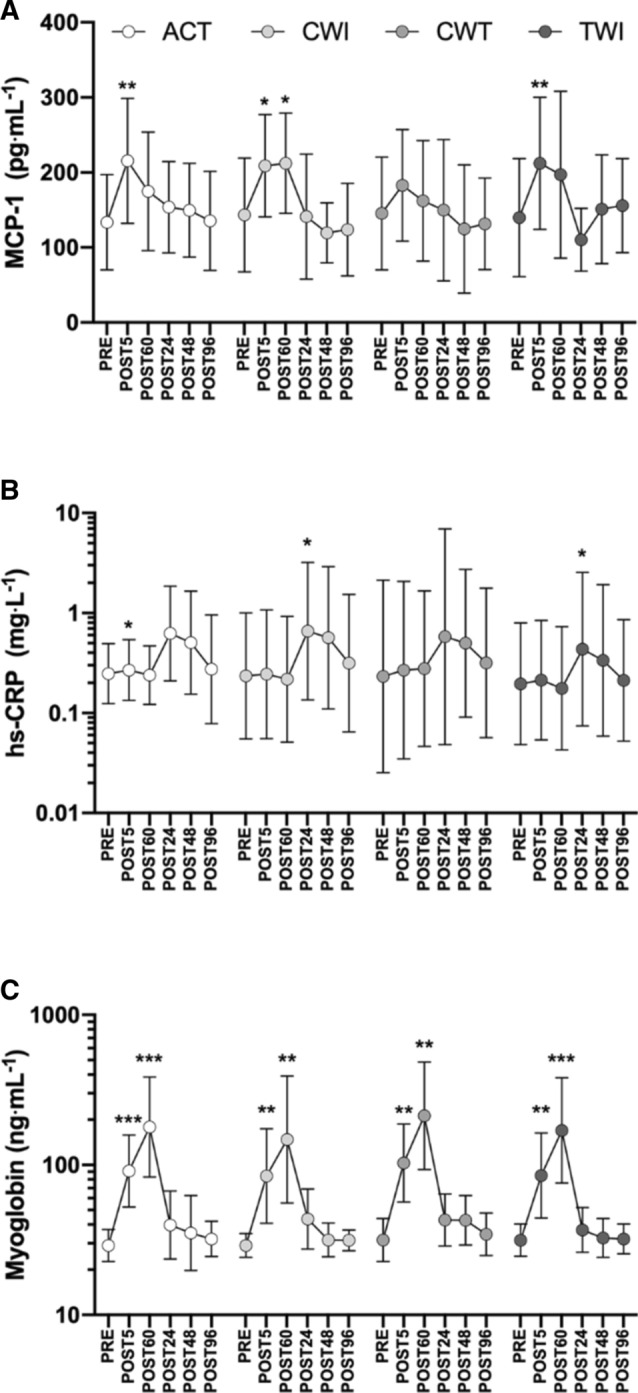
MCP-1 (**a**), hs-CRP (**b**), and myoglobin (**c**) responses to loading and recovery. Recovery interventions applied between POST5 and POST60; ACT, active recovery only, CWI, cold-water immersion, CWT, contrast water therapy and TWI, thermoneutral water immersion. Data are geometric mean and SD factors displayed on a log_10_ scale. No significant differences in biomarker responses were found between recovery interventions; however, effects of time are displayed for each marker as differences from PRE. *p < 0.05, **p < 0.01, ***p < 0.001

Two-way repeated-measures ANOVAs revealed no significant trial × time interactions in the blood biomarker responses (MCP-1, myoglobin, and hs-CRP) to the three water immersion methods compared to active recovery (Fig. 3). Trial × time interactions typically accounted for a very low percentage of total variance for all variables (< 3%). As expected, multiple time effects were observed between timepoints for each biomarker; for clarity, only differences from PRE are described. MCP-1 generally increased from PRE to POST5 and returned to baseline between POST60 and POST24 (Fig. 3a). hs-CRP generally increased between POST60 and POST24 and recovered to baseline between 48 and 96 h post-loading (Fig. 3b). Myoglobin increased from PRE to POST5 and continued to rise until POST60 (Fig. 3c). Myoglobin then decreased significantly from POST60 to POST24 and continued to recover until 48 h post-loading. No correlations were observed between biomarkers or biomarker responses.

### Exploratory descriptive analysis: variability of the immune response to a standardised loading protocol

We noted that the variability of immune and inflammatory responses to the loading protocol was high within individuals, within the portion of the trial that was standardised on all visits. Therefore, we *a posteriori* investigated the variability of the exercise-induced muscle damage, inflammatory, and leukocyte responses by calculating the intraclass correlation coefficient (ICC) for biomarker concentrations and leukocyte counts at PRE, POST5 and the response magnitude between the two timepoints (fold change). Creatine kinase (CK) and cortisol (COR) responses to exercise, previously reported in Ahokas et al (2019), were also included in the a posteriori analysis. The ICC model selected was a two-way, mixed-effects model with absolute agreement (Koo and Li 2016). ICC values with 95% confidence intervals are displayed in Tables 1 and 2. Two-way repeated-measures ANOVAs were also used to explore trial-order effects across these two timepoints (Supplementary Figure S1 and Supplementary Figure S2). Trial-order effects were evident for COR (Trial 1 mean > Trial 3 and 4, p < 0.01, Fig S1C), myoglobin (Trial 1 POST5 > Trials 2‒4, p < 0.01, Fig. S1F) and neutrophils (Trial 1 POST5 > Trial 3 and 4, p < 0.01, Fig. S2).

**Table 1:** Intraclass correlation coefficients for biomarkers measured at PRE and POST5 and the response magnitude from PRE to POST5 across all four trials. *, p < 0.05, **, p < 0.01, ***, p < 0.001 with ICC = 0 as reference

Variable	ICC pre	ICC post	ICC response magnitude
MCP-1	0.718*** (0.440‒0.914)	0.659*** (0.359–0.892)	0.139 (− 0.109 to 0.576)
CK	0.555*** (0.220–0.851)	0.338* (0.029–0.732)	0.065 (− 0.131 to 0.481)
hs-CRP	0.353* (0.043–0.878)	0.388** (0.071–0.762)	0.402** (0.084 to 0.771)
COR	0.322** (0.045–0.710)	0.556*** (0.232–0.848)	0.609*** (0.298 to 0.872)
Myoglobin	0.660*** (0.357–0.892)	0.376*** (0.075–0.746)	0.277*** (0.028 to 0.664)

**Table 2:** Intraclass correlation coefficients for white blood cells measured at PRE and POST5 and the response magnitude from PRE to POST5 across all four trials. *, p < 0.05, **, p < 0.01, ***, p < 0.001 with ICC = 0 as reference

Variable	ICC pre	ICC post	ICC response magnitude
Leukocytes	0.587*** (0.262‒0.864)	0.463*** (0.130‒0.806)	0.238 (− 0.036 to 0.657)
Lymphocytes	0.463** (0.139–0.804)	0.536*** (0.194‒0.843)	0.548*** (0.230–0.845)
Mixed cells	0.215 (− 0.059 to 0.643)	0.248 (− 0.046 to 0.673)	− 0.034 (− 0.234 to 0.408)
Neutrophils	0.659*** (0.354–0.893)	0.664*** (0.366–0.894)	0.240* (0.006–0.633)

## Discussion

The main finding of this study was that water immersion methods did not alter circulating muscle damage and inflammation biomarkers after high-intensity sprinting and jumping exercise. Therefore, the water immersion methods used in the present study were not shown to give any added benefits on the clearance of inflammation, nor did they attenuate muscle damage and inflammation responses assessed with circulating biomarkers.

A bout of high-intensity loading triggers a transient inflammatory response comprising augmented white blood cell counts and stimulation of pro- and anti-inflammatory cytokine production (Freidenreich and Volek 2012). The physiological stress caused by exercise acts as a major stimulus for muscle fibre hypertrophy, and efficient repair of muscles requires a well-coordinated and controlled inflammatory response (Peake et al. 2010). C-reactive protein (CRP) is an acute phase reactant that can be used to monitor inflammation episodes (Brouwer and van Pelt 2015). In the present study there were no differences in hs-CRP between recovery interventions, although CRP responded to and recovered from loading in all groups. Previous studies on CRP have reported either no difference (Ingram et al. 2009; Pointon et al. 2012) or decreased CRP levels after CWI compared to passive recovery (Brophy-Williams et al. 2011), and TWI (Ascensão et al. 2011). Physiological responses to water immersion methods have been suggested to be influenced by hydrostatic pressure and water temperature (Wilcock et al. 2006). The cold temperature of CWI is believed to enhance recovery by attenuating the inflammatory response to muscle-damaging exercise, as cold induces localised vasoconstriction (Mawhinney et al. 2017), which is suggested to reduce permeability of cellular, lymphatic, and capillary vessels, reducing fluid diffusion into the interstitial space (Eston and Peters 1999; Murray and Cardinale 2015). Furthermore, reduced fluid diffusion may assist in reducing acute inflammation and oedema (Cote et al. 1988; Yanagisawa et al. 2003; Stacey et al. 2010; Murray and Cardinale 2015). However, the results of previous studies (Pournot et al. 2011; Crystal et al. 2013; Broatch et al. 2014; de Andrade Bezerra et al. 2014) and the present study do not support these assumptions.

Exercise-induced muscle damage is triggered when individuals engage in new types of exercise, especially lengthening or eccentric contractions, which are conducted with a large range of motion. The mechanisms implicated in exercise-induced muscle damage, inflammation, and hypertrophy have been comprehensively reviewed elsewhere (Wackerhage et al. 2019). Blood levels of myofibre proteins such as myoglobin and CK are often used to quantify exercise-induced muscle cell injury (Warren et al. 1999). The present study did not observe any differences in myoglobin concentration between recovery interventions. Previously, CWI has been found to decrease blood myoglobin concentration compared to passive recovery (Bailey et al. 2007), TWI (Ascensão et al. 2011), and active recovery (Roberts et al. 2014). These results were found 30 min (Bailey et al. 2007), 60 min (Ascensão et al. 2011), and 2‒6 h (Roberts et al. 2014) after exercise. However, after 24‒48 h, there were no differences between CWI and passive recovery (Bailey et al. 2007) or TWI (Ascensão et al. 2011).

The cytokine monocyte chemoattractant protein-1 (MCP-1) is a sensitive marker of inflammation that increases immediately after muscle-damaging exercise (Peake et al. 2005). MCP-1 is necessary for the recruitment of monocytes for phagocytosis and facilitates repair of damaged myocytes (Chazaud et al. 2009; Lu et al. 2011). MCP-1 production is either constitutive or inducible by oxidative stress, cytokines or growth factors (Deshmane et al. 2009). To promote skeletal muscle repair, MCP-1 signalling up-regulates IGF-1 expression via intramuscular macrophages (Lu et al. 2011); indeed, MCP-1 deficient mice display impaired skeletal muscle regeneration (Shireman et al. 2007; Lu et al. 2011). Thus, accelerating return of MCP-1 concentrations to baseline after exercise may negatively impact training adaptation (Crystal et al. 2013). On the other hand, a reduction in MCP-1 release may be beneficial for short-term recovery, because it may inhibit the secondary damage arising as a result of inflammation following exercised-induced muscle damage (Crystal et al. 2013) and thus it could allow higher training frequency for possible better adaptation and performance during a tight competition season. However, in the present study MCP-1 concentration was unaffected by the recovery intervention. Only a few studies have investigated the effect of water immersion methods on MCP-1. In line with the present study, Crystal et al. (2013) did not find a difference in plasma MCP-1 concentration between CWI and passive recovery, but they observed a trend towards lower MCP-1 in the CWI group 6 h after exercise. Similarly, Peake et al. (2017) did not find a difference in MCP-1 expression between CWI and active recovery at 2, 24, and 48 h after exercise. These studies used different loading and recovery protocols compared to the present study. The present study and Peake et al. (2017) used 10 °C water temperature and 10 min immersion time in CWI. However, Crystal et al. (2013) used 5 °C water temperature and 20 min immersion time, resulting in higher cooling capacity. It has been suggested that CWI protocols of greater cooling capacity (i.e., of longer duration or colder water temperature) may exacerbate the inflammatory response in the circulation (Lee et al. 2012; White et al. 2014), because cold water imposes a compound stress on the body (White et al. 2014). Therefore, it seems that CWI does not affect MCP-1 expression even if the cooling capacity varies. In the future, the effects of water immersion methods on MCP-1 expression could be studied using team sport and endurance exercise protocols, because CWI has been found to promote the recovery of performance and clearance of blood myoproteins after exercise with higher metabolic demands and longer durations compared to this study (Ihsan et al. 2016). However, from the results of the present study, it seems like CWI does not accelerate the return of MCP-1 concentrations to baseline after muscle-damaging jumping and sprinting exercise.

In line with several previous studies (Pournot et al. 2011; Broatch et al. 2014; de Andrade Bezerra et al. 2014), we observed no differences in leukocytes and their subgroups (neutrophils, lymphocytes and mixed cells) between the four recovery methods. On the contrary, Stacey et al. (2010) found that after CWI, leukocyte and neutrophil counts were higher and lymphocyte counts were lower compared to both active and passive recovery. Stacey et al. (2010) used a similar recovery protocol in CWI (10 °C, 10 min) to both previous studies and the present study (10 °C, 10‒15 min) (Pournot et al. 2011; Broatch et al. 2014; de Andrade Bezerra et al. 2014). However, in the study by Stacey et al. (2010) the loading protocol (high-intensity cycling) was metabolically more demanding, which might explain the observed different response. One plausible explanation for the fact that the present study did not observe significant differences between recovery methods could have been the loading protocol. It may be considered whether the loading protocol was sufficiently stressful to highlight the differences between the recovery methods. Cortisol levels, previously reported in Ahokas et al (2019), did not increase after the loading protocol, suggesting that as expected stress caused by exercise was low. It is possible that the exercise protocol may not be demanding enough to induce high levels of inflammation, where differences in recovery methods would be measured. Nevertheless, the recovery protocol was designed to match possible recovery routines used by athletes, which is where the present study differs from previous studies, and is beneficial for external validity. It should also be taken into account, that in the present study the participants were physically active men, but not athletes. It has been noted previously that physiological responses may differ between high level athletes and active men (Ahtiainen et al. 2003).

To avoid the repeated bout effect of the inflammatory response to exercise that has been previously illustrated following muscle-damaging eccentric exercise bouts (McHugh 2003), the participants took part into familiarisation session and a randomised design was used. Nevertheless, post-hoc reliability and repeatability analyses of biomarker concentrations at timepoints that were reproduced in all four trials suggested that the reliability of biomarker responses to exercise loading was low. Applied in the present context, the ICC essentially represents a ratio of within-subject variation to between-subject variation (Weir 2005). Typically, ICCs below 0.5 have been defined as poor reliability (Koo and Li 2016), and in the present study only lymphocytes displayed ICCs for the response magnitude to exercise loading above this threshold. Cortisol, previously reported in Ahokas et al (2019), also displayed a satisfactory ICC for the response to exercise, but it is, however, notable that cortisol was also the only biomarker that did not increase significantly in response to loading. Moreover, trial-order effects were evident for myoglobin and neutrophils, indicative of a blunting in the exercise response across the four trials, particularly from trial 1. This suggests that the single familiarisation trial was not sufficient to account for the ‘repeated bout effect’ for these markers. If we were to hypothesise that low reliability of biomarker responses extends to timepoints after the different interventions in each trial, we could suggest that subtle influences of recovery measures on the rate of resolution of inflammation may have been masked by high intra-individual variability. Although main effects of time during recovery were detected, with many biomarkers displaying a clear time-course response after loading and recovery, it is possible that due to variability in exercise-induced responses, a greater number of participants may have been required to detect interaction effects between recovery interventions over time. This critique could be extended to previous studies that have aimed to address similar research questions, which typically recruit similar numbers of participants to the present study (Stacey et al. 2010; Crystal et al. 2013; Broatch et al. 2014). High-quality studies are needed to perform meta-analytical analyses. Meta-analytical analyses of multiple studies performed under similar conditions may help to account for low statistical power in individual studies; indeed recent meta-analytical findings suggest that CWI may accelerate resolution of post-exercise inflammation despite individual studies often showing no effects (Higgins et al. 2017). A strength of this study was the use of a repeated-measures crossover approach, which helped to mitigate the limitation of a small sample size, though to a lesser extent for markers with low ICCs. Furthermore, the water immersion methods were not compared only to passive recovery, but the purpose was to investigate the added benefit of the water immersion methods compared to standard recovery routines frequently used by athletes.

In conclusion, the results of this study support the hypothesis that water immersion methods do not accelerate recovery from loading exercise via anti-inflammatory effects when combined with an active recovery protocol. It is important to acknowledge that the exploratory descriptive analysis of the reliability of the exercise-induced response to a standardised loading protocol in the present study showed that there was a low reliability of biomarker responses to a standardised loading exercise bout, even after an initial familiarisation trial. Thus, future studies with higher numbers of participants, improved familiarisation to loading exercise, or meta-analytic analyses are needed to clarify this issue. Future studies should take into account potential adaptation effects when performing repeated loading exercise and ensure sufficient time for wash-out.

## Electronic supplementary material

Below is the link to the electronic supplementary material.

Supplementary material 1 (DOCX 670 kb)

## Data Availability

Data is available on request.

## Abbreviations

ACT: Active recovery only
CK: Creatine kinase activity
COR: Serum cortisol
CWT: Contrast water therapy
CWI: Cold-water immersion
hsCRP: High-sensitivity C-reactive protein
MCP-1: Monocyte chemoattractant protein-1
TWI: Thermoneutral water immersion
WBC: Leukocytes
