# Supplementary material for: Water immersion methods do not alter muscle damage and inflammation biomarkers after high-intensity sprinting and jumping exercise
Source: Eur J Appl Physiol. 2020 Sep 2;120(12):2625–34. doi: 10.1007/s00421-020-04481-8 (PMC7674333; doi:10.1007/s00421-020-04481-8)
Supplement: Supplementary file 1 — Supplementary material 1 (DOCX 670 kb) [file 421_2020_4481_MOESM1_ESM.docx]

**Supplementary material**

**Figure S1.** Biomarker data arranged by trial order across the two common time points repeated in each trial before intervention. Left panels show mean and SD (MCP-1), or geometric mean and SD factors displayed on a log_10_ scale; right panels display fold changes (MCP-1) or log_2_ fold changes in biomarker concentrations from pre to post-exercise for individuals across four repeated trials. Trial order effects were evident for cortisol (main effect of trial, p < 0.01, illustrated in panel C) and myoglobin (time × trial interaction, p < 0.001, significant trend towards blunting of the exercise response across trials, illustrated in panel F).

**
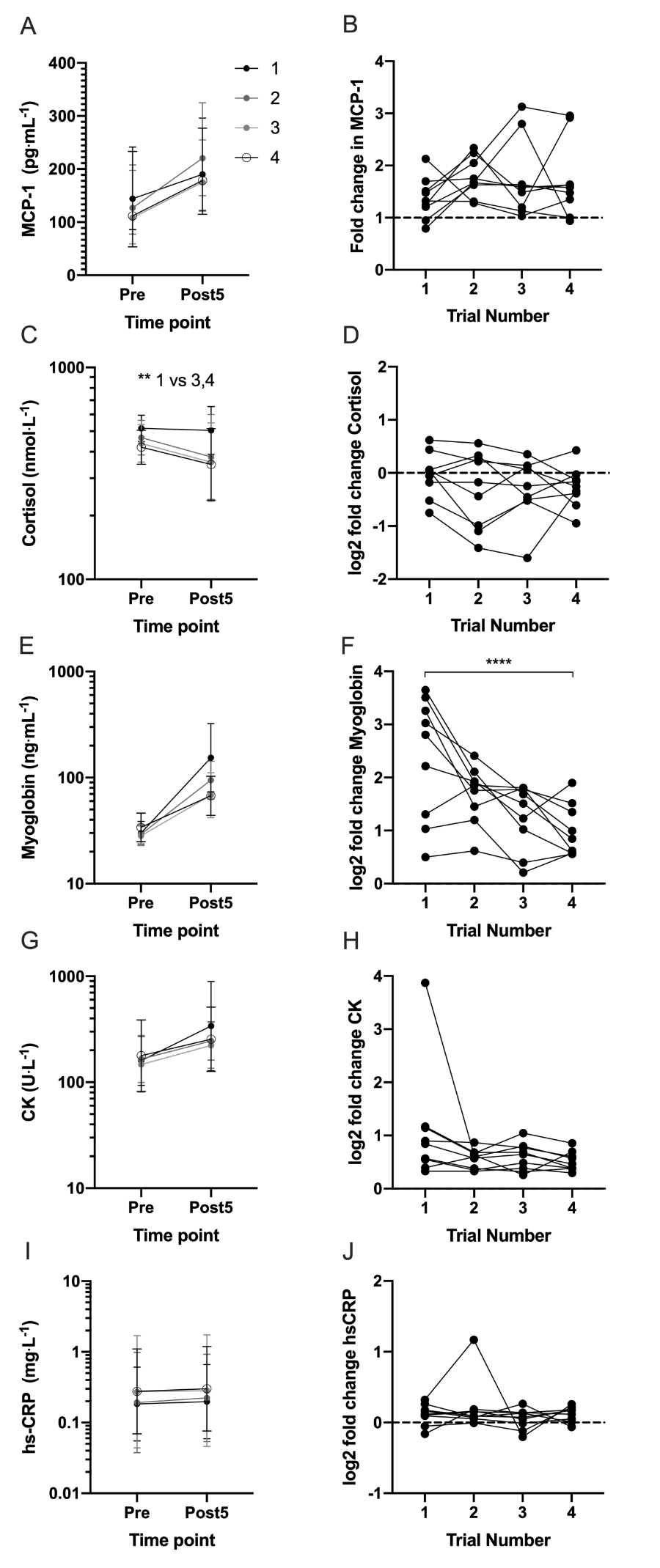
**

**
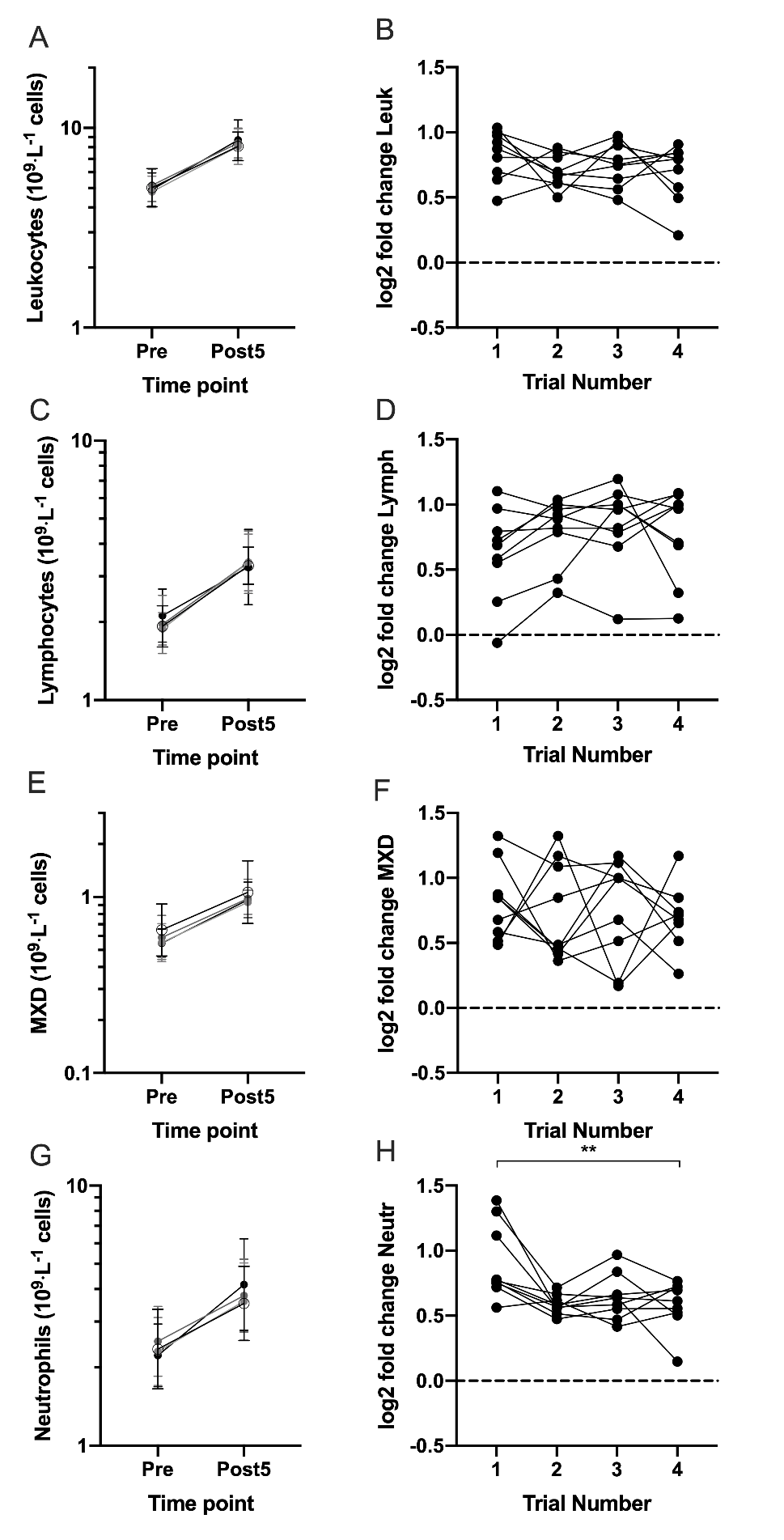
Figure S2:** White blood cell counts arranged by trial order across the two common time points repeated in each trial before intervention. Left panels show group mean and SD factors, displayed on a log_10_ scale; right panels log_2_ fold changes from pre to post for each participant across four repeated trials. A trial order effect occurred in neutrophils (time × trial interaction, p < 0.01, significant trend towards blunting of exercise response across trials)
